# Effect of beverage glucose and sodium content on fluid delivery

**DOI:** 10.1186/1743-7075-6-9

**Published:** 2009-02-20

**Authors:** Asker E Jeukendrup, Kevin Currell, Juliette Clarke, Johnny Cole, Andrew K Blannin

**Affiliations:** 1 School of Sport and Exercise Sciences, University of Birmingham, Edgbaston, Birmingham, UK

## Abstract

**Background:**

Rapid fluid delivery from ingested beverages is the goal of oral rehydration solutions (ORS) and sports drinks.

**Objective:**

The aim of the present study was to investigate the effects of increasing carbohydrate and sodium content upon fluid delivery using a deuterium oxide (D_2_O) tracer.

**Design:**

Twenty healthy male subjects were divided into two groups of 10, the first group was a carbohydrate group (CHO) and the second a sodium group (Na). The CHO group ingested four different drinks with a stepped increase of 3% glucose from 0% to 9% while sodium concentration was 20 mmol/L. The Na group ingested four drinks with a stepped increase of 20 mmol/L from 0 mmol/L to 60 mmol/l while glucose concentration was 6%. All beverages contained 3 g of D_2_O. Subjects remained seated for two hours after ingestion of the experimental beverage, with blood taken every 5 min in the first hour and every 10 min in the second hour.

**Results:**

Including 3% glucose in the beverage led to a significantly greater AUC 60 min (19640 ± 1252 δ‰ vs. VSMOW.60 min) than all trials. No carbohydrate (18381 ± 1198 δ‰ vs. VSMOW.60 min) had a greater AUC 60 min than a 6% (16088 ± 1359 δ‰ vs. VSMOW.60 min) and 9% beverage (13134 ± 1115 δ‰ vs. VSMOW.60 min); the 6% beverage had a significantly greater AUC 60 min than the 9% beverage. There was no difference in fluid delivery between the different sodium beverages.

**Conclusion:**

In conclusion the present study showed that when carbohydrate concentration in an ingested beverage was increased above 6% fluid delivery was compromised. However, increasing the amount of sodium (0–60 mmol/L) in a 6% glucose beverage did not lead to increases in fluid delivery.

## Introduction

One of the main aims of oral rehydration solutions (ORS) and sports drinks is to make the ingested fluid available to for use within the body as quickly as possible. Drinks designed for use in both ORS and sports nutrition contain a mixture of carbohydrate and electrolytes, with the main electrolyte being sodium.

Carbohydrates in ORS mainly come in the form of glucose, although they may also contain sucrose, maltodextrins or fructose. Increasing the amount of carbohydrate in an ingested beverage leads to a decrease in fluid delivery [1]. The increase in osmolality due to high carbohydrate concentrations leads to a net movement of water into the intestinal lumen causing a loss in the body water pool and may increase the effects of dehydration [2]. Previously it has been shown that a 6% CHO electrolyte solution leads to greater fluid delivery than a 15% glucose solution [3], but there was no difference shown in fluid delivery when a 6%, 8% and 10% glucose and fructose solution was compared [4].

Previous research has suggested that addition of sodium to ingested beverages will lead to increased fluid delivery [5] and a reduced plasma volume change during exercise indicating greater availability of fluid [6]. More recent research has suggested that sodium content may not be as important a factor as carbohydrate [7]. Indeed, increasing the sodium content of a 6% carbohydrate solution did not show any differences in intestinal water absorption [8]. However, this investigation employed the triple lumen technique which only measures a small section of the small intestine and does not take into account gastric emptying. Therefore, the triple lumen technique may not be representative of fluid availability to the whole body from an ingested beverage.

Deuterium oxide dilution is a relatively non invasive measure of fluid delivery. While it does not give a quantitative value of the amount of fluid absorbed as long as the amount and concentration of the tracer are kept the same the method can provide a measure of relative differences between ingested beverages [9]. Inclusion of D_2_O in an ingested beverage gives an integrative measure of gastric emptying and intestinal fluid absorption leading to fluid delivery. Studies which have compared different beverages using D_2_O [3,10] have found temporal D_2_O responses which would be expected [11].

The aim of the present study was to investigate the effects of increasing carbohydrate and sodium content upon fluid delivery using a deuterated water tracer.

## Methods

Twenty healthy male subjects took part in the study. The study was approved by the Ethics Committee of the School of Sport and Exercise Sciences at the University of Birmingham. Subjects completed a General Health Questionnaire and provided written consent to take part in the study.

All experimental trials took place after an overnight fast. Each subject undertook four trials each at least 7 days apart. The subjects were divided into two groups of 10, the first group was a carbohydrate group (CHO) (Age: 20 ± 1 y, Body Mass: 81.2 ± 7.5 kg) and the second a sodium group (Na) (Age: 21 ± 2 y, Body Mass: 83.6 ± 9 kg). The experimental beverages given to the CHO group were:

**G0**: Water + 20 mmol/L sodium

**G3**: 3% Glucose + 20 mmol/L sodium

**G6**: 6% Glucose + 20 mmol/L sodium

**G9**: 9% Glucose + 20 mmol/L sodium

The Na group ingested the following beverages:

**Na0**: 6% Glucose

**Na20**: 6% Glucose + 20 mmol/L sodium

**Na40**: 6% Glucose + 40 mmol/L sodium

**Na60**: 6% Glucose + 60 mmol/L sodium

Glucose was obtained from Cerestar (Manchester, UK) and sodium from Sigma-Aldrich (Gillingham, UK). All trials were conducted in a randomised order.

Subjects arrived in the laboratory between 7 and 9 am. On arrival in the laboratory subjects were asked to empty their bladder before nude body mass was recorded (Champ II, Ohaus UK, Leicester, UK). Thereafter a Teflon catheter (Quickcath, Baxter, Norfolk, UK) was inserted into the antecubital vein for blood sampling.

Subjects remained seated for 20 min before a resting blood and saliva sample was taken. A 550 ml bolus of the experimental beverage was given containing 3.00 g of deuterated water (99.9% atom % deuterium oxide, Sigma Aldrich, St Louis USA), this was then followed by a further 50 ml of the experimental beverage which was used to swill out the mouth and ensure all deuterium oxide was ingested.

A 5 ml blood sample was taken every five minutes following ingestion of the experimental beverage for the first hour and every 10 min for the second hour. Therefore, subjects remained at rest for a total of 120 min. At all time point's blood was stored in tubes containing 0.054 ml K_3_EDTA (Becton Dickinson, Plymouth, UK). All EDTA tubes were centrifuged at 3200 g for 10 min and the plasma stored in a glass vial which was stored at -70°C.

Plasma D_2_O enrichment were analysed using the GasBench II (Thermo Electron, Bremen, Germany) – isotope ratio mass spectrometry (Finnigan, Delta XP, Bremen, Germany). Briefly, 200 μl of the aqueous sample was transferred into a vacutainer (Labco, High Wycombe, England). A platinum catalyst (Thermo Electron, Bremen, Germany) was added and the vacutainer flushed with a 2% H_2 _in Helium for 5 min gas followed by a 40 min equilibration period then followed where the hydrogen isotopes in the aqueous solution exchanged with hydrogen ions in the headspace. A sample of the headspace gas was then injected into an Isotope Ratio Mass Spectrometer (IRMS) (Thermo Electron, Bremen, Germany). The average of the last 9 measurements was used as the average for the sample. The isotopic enrichment is expressed as δ‰ against the international water standard Vienna Standard Mean Ocean Water (VSMOW). The CV of this measurement is 0.27%.

Non linear regression was employed to calculate the half time (T_1/2_), time to plateau (TTP) and plateau enrichment (PE) of the enrichment curve (GraphPad Prism, GraphPad software, San Diego, US). The non linear regression equation used was:

Y = Ymax.(1-e^(-*K*.*t*)^)

Where Y = plasma D_2_O enrichment, K = a constant, and t = time.

In both experiments plasma deuterium oxide enrichment were analysed using a two way (time and treatment) ANOVA for repeated measures. Area under the curve for the first 60 min (AUC 60 min), T_1/2_, time to plateau and plateau enrichment were analysed using a repeated measures ANOVA. All post hoc tests were Tukeys HSD. Significance was set at p < 0.05. All data analysis was conducted using SPSS version 12.

## Results

The glucose group showed a quick rise in plasma deuterium oxide enrichment before reaching a plateau. While not significantly different to each other there was a trend for the time to plateau to increase with increasing carbohydrate concentration (GO = 34 ± 7 min, G3 = 35 ± 10 min, G6 = 43 ± 13 min, G9 = 51 ± 15 min), all reached a plateau enrichment of similar value (G0 = 434 ± 113 δ‰ vs. VSMOW, G3 = 458 ± 96 δ‰ vs. VSMOW, G6 = 398 ± 106 δ‰ vs. VSMOW, G9 = 360 ± 55 δ‰ vs. VSMOW).

**Figure 1:**
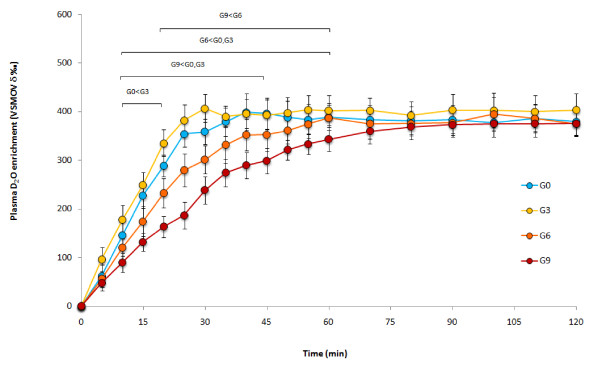
**D_2_O enrichment over time after ingesting 4 different glucose beverages**. Statistical differences (P < 0.05) are indicated.

The glucose group showed a significant effect of trial (F_3,27 _= 37.250, P < 0.001), time (F_18,162 _= 88.973, P < 0.001) and an interaction between trial and time (F_54,486 _= 5.386, P < 0.001). Including 3% glucose in the beverage led to significantly greater plasma deuterium enrichment than having no carbohydrate between 20 and 30 min after ingestion. When carbohydrate content was increased to 6% there was lower plasma D_2_O enrichment than the 3% and 0% carbohydrate beverages between 10 and 45 min after ingestion. Further increasing the carbohydrate content to 9% led to lower plasma deuterium enrichment than a 3% and 0% beverage between 10 and 60 min after ingestion, and also had lower plasma deuterium enrichment than a 6% beverage between 20 and 60 min after ingestion (Figure 1).

There was a significant effect of trial for T_1/2 _(F_3,27 _= 17.528, P < 0.001). Trials G0 (11 ± 2 min) and G3 (9 ± 1 min) had the quickest T_1/2 _but were not different from each other and trial G6 (15 ± 2 min) was quicker than G9 (24 ± 3 min) (Table 1). There was a significant effect of trial for AUC 60 min (F_3,27 _= 65.861, P < 0.001). Trial G3 showed the greatest AUC 60 min (19640 ± 1252 δ‰ vs. VSMOW.60 min), with G0 (18381 ± 1198 δ‰ vs. VSMOW.60 min) having a greater AUC 60 min than G6 (16088 ± 1359 δ‰ vs. VSMOW.60 min) and G9 (13134 ± 1115 δ‰ vs. VSMOW.60 min), G6 had a significantly greater AUC 60 min than G9 (Table 1).

**Table 1:** D_2_O enrichment characteristics with 4 different glucose beverages

	**G0**	**G3**	**G6**	**G9**
**T 1/2 (min)**	11 ± 2^bc^	9 ± 1^bc^	15 ± 2^c^	24 ± 3
**AUC (δ‰ vs. VSMOW.60 min)**	18381 ± 1198^bc^	19640 ± 1252^abc^	16088 ± 1359^c^	13134 ± 1115
**PE (δ‰ vs. VSMOW)**	434 ± 113	458 ± 96	398 ± 106	360 ± 55
**TTP (min)**	34 ± 7	35 ± 10	43 ± 13	51 ± 15

Half time (t1/2), area under the curve for first 60 min (AUC), plateau enrichment (PE) and Time to Plateau (TTP) with no glucose (G0), 3% glucose (G3), 6% glucose (G6) and 9% glucose (G9). All drinks contained 20 mmol/L sodium. Significant differences are indicated with a, b and c. a indicates different from G0, b indicates different from G6, c indicates different from G9. (P < 0.05)

**Figure 2:**
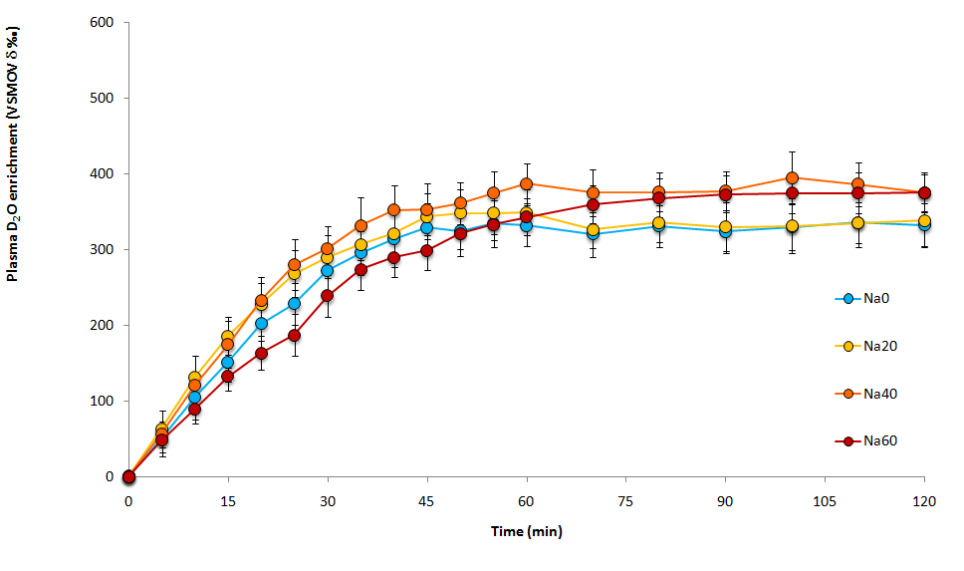
**D_2_O enrichment over time after ingesting 4 different sodium beverages**. There were no statistical differences.

The sodium group also had an initial increase in plasma deuterium oxide enrichment before reaching a plateau (Figure 2). While not significantly different from each other there was a trend for a decrease in time to plateau with increasing sodium content (Na0 = 23 ± 3 min, Na20 = 19 ± 3 min, Na40 = 18 ± 2 min, Na60 = 16 ± 3 min), all had similar plateau enrichments (Na0 = 352 ± 17 δ‰ vs. VSMOW, Na20 = 356 ± 14 δ‰ vs. VSMOW, Na40 = 348 ± 15 δ‰ vs. VSMOW, Na60 = 354 ± 18 δ‰ vs. VSMOW) (Table 2).

**Table 2:** D_2_O enrichment characteristics with 4 different sodium beverages

	**Na0**	**Na20**	**Na40**	**Na60**
**T 1/2 (min)**	16 ± 2 min	13 ± 2 min	12 ± 1 min	11 ± 2
**AUC (δ‰ vs. VSMOW.60 min)**	13843 ± 785	15054 ± 674	15162 ± 467	15810 ± 719
**PE (δ‰ vs. VSMOW)**	352 ± 17	356 ± 14	348 ± 15	354 ± 18
**TTP (min)**	23 ± 3	19 ± 3	18 ± 2	16 ± 3

Half time (t1/2), area under the curve for first 60 min (AUC), plateau enrichment (PE) and Time to Plateau (TTP) with no sodium (Na0), 20 mmol/L sodium (Na20), 40 mmol/L sodium (Na40) and 60 mmol/L sodium (Na60) in a 6% glucose solution. There were no statistical differences between trials.

The sodium group failed to show any differences between the trials for plasma D_2_O enrichment (F_3,27 _= 1.719, P = 0.187) (Figure 2). While Na60 had the quickest T_1/2 _(11 ± 2 min) compared to Na40 (12 ± 1 min), Na20 (13 ± 2 min) and Na0 (16 ± 2 min), these were not significantly different from each other (F_3,27 _= 2.119, P = 0.121). Similarly Na60 had the greatest AUC 60 min (15810 ± 719 δ‰ vs. VSMOW.60 min) compared to Na40 (15162 ± 467 δ‰ vs. VSMOW.60 min), Na20 (15054 ± 674 δ‰ vs. VSMOW.60 min) and Na0 (13843 ± 785 δ‰ vs. VSMOW.60 min) but again were not significantly different from each other (Table 2).

## Discussion

The present study investigated the effect of increasing the amounts of glucose and sodium in ingested beverages on fluid delivery. A deuterium oxide tracer was incorporated in the test beverages and plasma deuterium oxide enrichment was used as a measure of fluid delivery.

Increasing the glucose content of the beverage above 6% decreased fluid delivery compared to water, whereas sodium content in the range investigated did not affect fluid delivery.

The present study shows that increasing the carbohydrate content of a beverage above 6% can lead to a decrease in fluid delivery compared to water. While previously a 15% glucose solution [3] and a 20% carbohydrate solution [12] have been shown to cause a slowing of the accumulation of deuterium oxide in plasma following ingestion of the beverage, this is the first study which has showed that a 6% carbohydrate solution can slow the accumulation of D_2_O in plasma. Davis *et al*. [4] did not report any differences in the appearance of D_2_O in plasma when comparing 6%, 8% and 10% glucose and fructose beverages with water. A possible reason for the discrepancies between these studies is the inclusion of fructose in the beverages of Davis *et al*. [4]. Fructose is absorbed by GLUT5 in the intestinal cell membrane [13], whereas glucose is absorbed by SGLT1 [14]. The inclusion of these multiple transportable carbohydrates can lead to a reduction of the inhibiting effect of hyperosmolality on fluid absorption [15].

As the use of a D_2_O tracer is an integrated measure of gastric emptying and intestinal absorption either of these could be the site of the decreased fluid delivery seen with the ingestion of the 6% and 9% glucose beverages. Upon ingestion of the beverage it first enters the stomach. A 20% carbohydrate solution was shown to empty slower from the stomach than a 6% carbohydrate solution [12]. Concentrations of carbohydrate less than 10% have also been shown to slow gastric emptying, with a 5% glucose beverage having a slower gastric emptying when compared to water alone, and a 10% glucose beverage slowing gastric emptying even further [1]. One study has reported that carbohydrate concentrations less than 10% do not affect gastric emptying [16]. It is possible that the 6% and 9% carbohydrate beverages in the present study slowed gastric emptying although this is not clear.

Once the ingested beverage has been emptied from the stomach it enters the small intestine. The proximal section of the small intestine, the duodenum is the most water permeable section of the small intestine. Water is absorbed down an osmotic gradient in the duodenum, therefore when water is compared to a carbohydrate beverage water leads to greater fluid absorption in the duodenum as it causes a greater osmotic gradient [17]. The increased carbohydrate content in the 6% and 9% beverages may have decreased fluid absorption in the duodenum compared to water [15].

As the ingested beverage continues down the small intestine to the jejunum more solute is absorbed [17]. The absorption of glucose by SGLT1 in the small intestine is directly coupled with the absorption of 2 sodium molecules and approximately 300 water molecules [18]. Therefore fluid can be absorbed against a concentration gradient. This may explain why the 3% glucose beverage led to greater fluid delivery than the water beverage. The absorption of glucose in the jejunum will lead to increased fluid absorption, both in the transcellular route of SGLT1 and through paracellular pathways as SGLT1 has been shown to increase the permeability of the intestinal tight junctions [19].

The present study did not show any effect of increasing sodium concentration on fluid delivery. It has been shown that when 25 mmol/L sodium chloride solution was ingested that the decrease in plasma volume seen during exercise was lowered compared to water alone, suggesting that fluid delivery was increased [6]. The present study suggests that when carbohydrate is included in a beverage increasing the amount of sodium does not increase fluid absorption. Gisolfi *et al*. [8] investigated the absorption of fluid from a 6% glucose beverage containing either 0, 25 or 50 mmol/L and found that there was no difference in fluid absorption. Similarly, during exercise increasing the amount of sodium in carbohydrate beverages ingested during exercise did not lead to an increase in fluid absorption [20]. Therefore, the presence of 6% glucose in the beverages in the present study may have masked any effect that sodium has upon fluid delivery.

Despite no effect of sodium on fluid delivery it might still be useful to include sodium into carbohydrate electrolyte beverages. Including sodium in an ingested beverage leads to an increase in palatability [21]. Palatability is an important factor in increasing voluntary drinking [22], which may be useful in situations such as during prolonged exercise in hot conditions as the fluid intake may enable a lower core body temperature to be maintained when compared to no fluid and prevent a decrease in performance [23]. Sodium is also important for rehydration after a period of dehydration as sodium helps with fluid retention [24].

Although deuterium oxide tracers have been used extensively to measure total body water (TBW), the use of this tracer to measure fluid delivery has received relatively little attention in the literature. Studies have successfully employed a deuterium oxide tracer to compare fluid delivery between drinks [3,12,25,26]. The advantages of the technique are that it is relatively non invasive and provides an integrated measure of the effects of gastric emptying and intestinal absorption on fluid delivery. One suggested disadvantage to the technique is that it is not able to measure net fluid absorption [2]. The results of these studies should always be interpreted with caution. The data presented here may not reflect absorption per se. This would require an assumption that steady state conditions exist for extra and intra cellular fluid volumes; and this assumption is not always valid. This would have been a particular concern in the sodium trials in this study. If increasing beverage sodium commensurately expanded plasma volume, one could have increased water absorption from the gut without a parallel increase in plasma D_2_O enrichment. To a lesser extent, a similar concern applies to the glucose trials. As insulin-mediated glucose uptake will promptly move absorbed glucose into the intracellular space, it will be followed by water, again creating a non-steady state dynamic during the period that the data are collected. The exact impact of these fluid shifts is unknown and therefore results have to be interpreted with caution.

In conclusion the present study showed that when carbohydrate concentration in an ingested beverage was increased above 6% fluid delivery was compromised. Sodium content (0–60 mmol/L) in a 6% glucose beverage did not lead to increases in fluid delivery.

## Competing interests

The authors declare that they have no competing interests.

## Authors' contributions

AJ was the principle investigator, managed the project and finalized the paper. KC performed the experimental trials and analyses and drafted the first manuscript. JC helped in the daily running of the trials in the laboratory. JC helped in the daily running of the trials in the laboratory. AB provided technical assistance with the deuterium oxide analysis. All authors read and approved the final manuscript.
